# Effects of Caller Characteristics on Auditory Laterality in an Early Primate (*Microcebus murinus*)

**DOI:** 10.1371/journal.pone.0009031

**Published:** 2010-02-03

**Authors:** Lisette M. C. Leliveld, Marina Scheumann, Elke Zimmermann

**Affiliations:** 1 Institute of Zoology, University of Veterinary Medicine Hannover, Hannover, Germany; 2 Center for Systems Neuroscience, Hannover, Germany; L'université Pierre et Marie Curie, France

## Abstract

**Background:**

Auditory laterality is suggested to be characterized by a left hemisphere dominance for the processing of conspecific communication. Nevertheless, there are indications that auditory laterality can also be affected by communicative significance, emotional valence and social recognition.

**Methodology/Principal Findings:**

In order to gain insight into the effects of caller characteristics on auditory laterality in the early primate brain, 17 gray mouse lemurs were tested in a head turn paradigm. The head turn paradigm was established to examine potential functional hemispheric asymmetries on the behavioral level. Subjects were presented with playbacks of two conspecific call types (tsak calls and trill calls) from senders differing in familiarity (unfamiliar vs. familiar) and sex (same sex vs. other sex). Based on the head turn direction towards these calls, evidence was found for a right ear/left hemisphere dominance for the processing of calls of the other sex (Binomial test: p = 0.021, N = 10). Familiarity had no effect on the orientation biases.

**Conclusions/Significance:**

The findings in this study support the growing consensus that auditory laterality is not only determined by the acoustic processing of conspecific communication, but also by other factors like the sex of the sender.

## Introduction

In the last hundred years cerebral laterality in the processing of language has received much attention and studies have found support for the existence of a left hemisphere dominance in the processing of language in humans (e.g. [1]–[3]). This predisposition of the left hemisphere for the processing of language was suggested to arise from a pre-linguistic advantage of the left hemisphere for processing information with a high temporal precision [2]), whereas the right hemisphere has an advantage for pitch perception (e.g. [4], [5]). However, there are several indications that other factors such as communicative significance [6], [7], emotional valence [8], [9], and familiarity with the speaker [9], [10], affect lateralized auditory processing.

The assumption that the lateralized processing of conspecific communication is unique to humans [11], is challenged by findings of left hemisphere dominance in the processing of conspecific communication sounds in other animal species, such as rhesus macaques [12]–[15], Japanese macaques [16]–[19] and sea lions [20]. Exceptions to this were reported in vervet monkeys [21] and Barbary macaques [22]. This left hemisphere dominance was, like in humans, also explained by its specialization for processing temporal cues (e.g. [13], [14], [23]).

However, also in non-human animals there are indications that auditory laterality of conspecific communication is affected by factors, such as communicative significance, emotional valence, and familiarity with the sender. First, studies on Japanese macaques [16]–[18], mice [24] and raptors [25] have shown that a communicative significance, achieved through exposure to calls in a meaningful context, is essential for establishing a left hemisphere dominance in the processing of these calls.

Second, there are studies in non-human animals that found an effect of emotional valence on auditory laterality. Sounds of negative emotional valence were found to be processed with a right hemisphere dominance in dogs [26] and Campbell's monkeys [9], but with a left hemisphere dominance in male mouse lemurs [27].

Third, visual perception of familiar conspecifics is found to be lateralized to the right hemisphere in several vertebrate species, such as domestic fowl (e.g. [28], [29]), quails [30], and sheep [31]. In contrast, in auditory perception only a few studies tested for lateralized processing of familiar conspecifics (here referred to as ‘familiarity-to-sender effect’). In a study on starlings, George et al. [32] found that the processing of songs of familiar conspecifics was lateralized to the right hemisphere, which suggests a similar lateralized processing of familiar conspecifics in auditory and visual modalities. However, the findings in auditory laterality are discussed controversially, because also a left hemisphere dominance was found in horses [33] and in zebra finches [34].

Since both humans and several non-primate species show this ‘familiarity-to-sender effect’, one would expect to find it also in non-human primates. Indeed, recently a voice recognition region has been identified in the primate brain, located in the middle of the anterior superior-temporal plane [35]. This suggests that voice recognition in non-human primates relies on similar neural substrates as in humans. Moreover, many primate species have shown to be able to discriminate between familiar and unfamiliar individuals, based on their vocalizations (e.g. [36], [37]). However, only a few studies have focused on the ‘familiarity-to-sender effect’ on the auditory lateralization in primates and with contradicting results. Recently, Campbell's monkeys were found to have a left hemisphere dominance for the processing of calls of familiar senders, but not of unfamiliar senders [9]. However, in vervet monkeys no effect of familiarity-to-sender was found on auditory laterality [21]. Because of these highly variable findings on lateralized auditory processing in humans, non-human primates and other vertebrate species, there is a need for more studies on the ‘familiarity-to-sender effect’ on auditory laterality.

Another sender characteristic that could have an influence on the lateralized processing of communication sounds, is its sex, here referred to as the ‘sex-of-sender effect’. To our knowledge, no study has so far compared the lateralized auditory processing of sounds of male and female senders in non-human animal listeners, and only one study tested the ‘sex-of-sender effect’ in humans. In humans, Landis et al. [38] reported a right ear advantage for the recognition of female voices and a left ear advantage for the recognition of male voices, indicating that the sex of the sender could affect the lateralized processing of the sound, at least in humans. In non-human animals, a ‘sex-of-sender effect’ on auditory laterality can be expected, since calls from males and females should be of different communicative significance to the listener, especially in the context of courtship. Indeed, lateralized behavior has been found in courtship approach, but with contradicting findings, since male black winged silts prefer to use the left eye in courtship behavior [39], whereas males in poeciliid fish approach females with a right eye preference [40].

Many of the studies that explored auditory laterality in non-human animals have used a so-called head turn paradigm [12]. In this paradigm a sound is played back to a subject from a loudspeaker that is placed 180 degrees behind the subject. It is assumed that turning one ear towards the source of the sound causes an increase in the intensity of the signal at that ear and, since each ear projects to the contralateral hemisphere, an auditory-input bias to the contralateral hemisphere [13]. Hence, turning the right ear towards the sound indicates a left hemisphere dominance for the processing of this sound, whereas turning the left ear indicates a right hemisphere dominance for the processing of this sound.

To gain insight into the evolution of auditory lateralization in primates, we investigated for the first time the effect of caller characteristics (familiarity and sex) on the lateralized auditory processing of communication calls in an early primate brain. Our model species is the gray mouse lemur (*Microcebus murinus*), a small bodied nocturnal prosimian species, endemic to Madagascar. It is suggested to represent the most ancestral primate condition [41]. Mouse lemurs have an elaborate vocal repertoire with both low frequency and ultrasonic communication calls [42]. In a previous study on auditory laterality in this species Scheumann and Zimmermann [27] already found that auditory laterality in gray mouse lemurs was affected by the sex of the listener (‘sex-of-receiver effect’) and by emotional valence, since they found that males (but not females) showed a left hemisphere dominance for the processing of conspecific communication calls of negative emotional valence (but not for calls of positive emotional valence). This species is therefore ideal to further explore the mechanisms that affect the lateralized auditory processing of conspecific communication calls. In the study by Scheumann and Zimmermann [27] only calls of unfamiliar female senders were used as playback stimuli. We hypothesized that the effects of call type and of the sex-of-receiver might have been influenced by the identity and sex of the senders in this previous study. Thus, in the present study we first explored the effects of the sex-of-receiver and call type, whilst controlling for the caller characteristics. Second, we explored whether caller characteristics (familiarity-to-sender and sex-of-sender) affect auditory laterality.

The head turn paradigm was used to study lateralized auditory processing on the behavioral level. In order to test for effects of sex-of-receiver and call type, we studied the orienting asymmetries in both male and female gray mouse lemurs in response to playbacks of two conspecific call types, the tsak call (used in agonistic contexts) and the trill call (used in social cohesion contexts). Both call types were found to be distinctive by caller [43], enabling the receiver to individually recognize the sender. Sex differences in the call structure have been found in the tsak call (unpublished results), but have not been studied yet in the trill call. In order to test for the effects of familiarity-to-sender and sex-of-sender, the present playback paradigm includes calls (trill and tsak) from three different senders; unfamiliar males, unfamiliar females and familiar, same sex conspecifics (cage mate).

**Figure 1:**
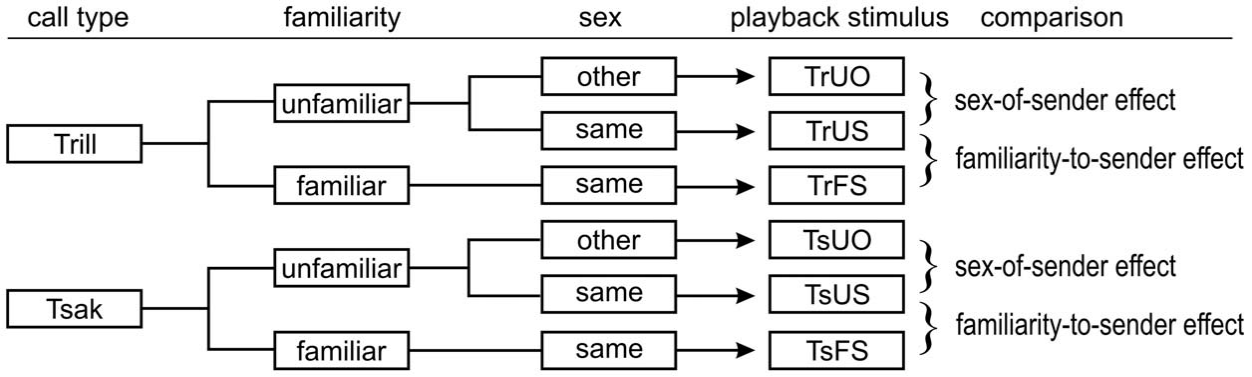
Schedule of the playback stimuli. This schedule shows how the playback stimuli differ from each other in call type, familiarity and sex, and how they are compared to test the effects of familiarity-to-sender and sex-of-sender.

Thus, we explored four effects on auditory laterality: Sex-of-receiver, call type, familiarity-to-sender and sex-of-sender. In order to test for an effect of sex-of-receiver we compared the responses of male and female subjects. In order to test for emotional valence, we compared the responses of the subjects to trill calls (positive emotional valence) with the responses to tsak calls (negative emotional valence). In order to test for a familiarity-to-sender effect, we compared the responses of the subjects to calls of familiar, same sex, conspecifics (FS) with the response to calls of unfamiliar, same sex conspecifics (US; Figure 1). In order to test for sex-of-sender effect, we compared the responses of the subjects to calls of unfamiliar, other sex, conspecifics (UO) with the response to calls of unfamiliar, same sex, conspecifics (US; Figure 1). Based on the literature we expect to find that caller-specific information (familiarity and sex) coded in the calls affects the auditory laterality.

## Materials and Methods

Our research adheres to the national guidelines of the German Society of Primatology (GfP) for research on non-human primates and was approved by the State Capital Hannover, Department of Law and Order, Industry and Veterinary Affairs (approval date: 24 March 2003; number: 42500/1H). Our research is in accordance with the recommendations of the Weatherall report, “the use of non-human primates in research”, since only non-invasive, behavioral studies were performed. The subjects were tested in a familiar test room, without any apparent stressors present. There was no physical contact between the experimenter and the subjects.

### Subjects

We tested 17 gray mouse lemurs (12 males, five females) of our breeding colony, housed in the animal facility of the Institute of Zoology, University of Veterinary Medicine Hannover (for details of housing conditions see [44]). All subjects were born in captivity. Their age ranged from nine months to eight years. 15 subjects were housed together with one or two other individuals of the same sex at the time of testing. Two subjects had been separated from their (same sex) cage mate shortly before the start of the experiment.

### Experimental Set-up

**Figure 2:**
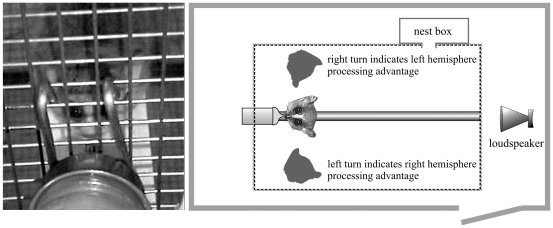
Experimental set-up (right) and defined head position (left). (adapted from Scheumann and Zimmermann, 2008).

Each mouse lemur was tested alone in a test cage (Ebecco stainless steel cage for marmosets, 80 cm ×87 cm ×50 cm) in a sound-attenuated chamber. The cage was equipped with two wooden bars, a nest box and a bottle with banana-peach juice. A loudspeaker was placed 180° on the opposite side of the nipple of the juice-bottle (Figure 2). To control for an effect of the nest box, it was placed either on the right (eight subjects) or the left (nine subjects) side of the cage. The playback stimuli were played back using the software NiDisk 1.33 by a Toshiba laptop equipped with a D/A converter card (National Instruments). The laptop was connected via an amplifier (Pioneer a-337) to a high frequency loudspeaker (Panasonic Leaf Tweeter EAS-Th400A, frequency range: 2–70 kHz). Subjects' behavior was videotaped using a digital camcorder (Sony DR-TRV 22E PAL mini DVD/Sony DCR-SR35E, Nightshoot). When using the Sony DR-TRV 22E PAL, the camera was linked to the tape output of a U-30 bat detector (Ultra Sound Advice) as external microphone. The camera was connected to a monitor outside the chamber where the experimenter sat and observed the subjects.

### Playback Stimuli

The playback stimuli were created from calls that were recorded from captive gray mouse lemurs of our breeding colony. Playback stimuli differed on three different levels: (1) call type (trill call vs. tsak call), (2) familiarity of the sender (familiar vs. unfamiliar), and (3) sex of the sender (same sex vs. other sex), creating six categories of playback stimuli (Figure 1). An unfamiliar sender is defined here as a conspecific that was never housed in the same room at the same time as the subject. A familiar sender is here defined as a conspecific that was housed in the same cage as the subject at the time of testing, or had been separated from the subject no longer than six months before the testing started. Due to these specific requirements concerning the sender of the acoustic stimulus, we created a different set of playback stimuli set for each subject.

An experimental trial consisted of the presentation of a playback stimulus. Each playback stimulus consisted of a sequence of three sounds separated by a constant interval of 3600 ms. The duration of the tsak call sequence was standardized to the duration of the trill call (of each playback category, for each subject) as the longest continuous sound element. All acoustic stimuli were diffused with a sound pressure level of 75±1 dB at a distance of 1 m (RMS measurement, Brüel und Kjær Measuring Amplifier Type 2610).

### Procedure

Each experiment was conducted at the beginning of the activity period of each subject. For the experiment a subject was removed from its home cage and placed in a new nest box, which was then attached to the test cage in a sound-attenuated chamber. During the experiment subjects were able to drink juice from the bottle through licking on the nipple of the bottle. The experiment started as soon as the door to the sound attenuated chamber was closed, to rule out any influence of the experimenter.

We habituated each subject to the experimental set-up and the experimental procedure. We defined a subject as habituated when it licked on the nipple of the bottle within the first five minutes of the experiment. When a subject reached the habituation criterion, we conducted the first test at one of the next days.

In the test, we started a playback stimulus when the subject was sitting in a defined position, meaning that it was licking on the nipple of the bottle while keeping its head straight and its hands on the wooden bar. Thereby, the loudspeaker was positioned 180° behind the subject. Within one test, three playback stimuli were played back to the subject in a random order, with a minimum inter-stimuli interval of five minutes. If the test could not be finished in more than two hours, we tested the remaining acoustic stimuli of this test on a separate day. Tests were separated by two to four days. A minimum of two days ( =  two tests) was needed to expose the subject to all six stimuli. Each animal was exposed to each playback stimuli three times ( =  three sessions). The sessions were separated by a minimum of seven days.

### Data and Video Analysis

When the test were videotaped using the Sony DR-TRV 22E PAL, we digitized all video tapes using InterVideo WinDVD creator 2. When tests were recorded using Sony DCR-SR35E, the already digital files were transferred to an external hard disk. We conducted a frame by frame analysis (25 frames/second) in Interact 3.1. (Mangold International GmbH). We determined the exact time (frame) that the playback was started, using Music Maker Deluxe 2005 Version 10.0 (Music Editor 2.01, Magix AG). This time point was transferred manually to Interact 3.1. We analyzed all experimental trials with regard to the head position at the start of the playback stimulus. Since sometimes subjects did not turn their head in response to the first sound of a playback stimulus, but to the second or third, we determined also the head position at the onset of the second or third sound. To test for orientation biases ( =  first head turns towards a playback stimulus), we selected for further analyses all trials in which the head criterion ( =  the subject was licking on the nipple of the bottle while keeping its head straight and its hands on the wooden bar) was fulfilled.

For the selected trials, we analyzed the head turn direction of the first head turn during the presentation of a sound, head turns during the intercall-intervals were not included. For each trial we scored the following head turn responses: no response – the subject did not turn its head more than 90° to either of the two sides within the stimuli playback, right turn – the subject turned its head more than 90° to the right side, left turn – the subject turned its head more than 90° to the left side.

To assess inter-observer reliability, a naïve person coded 20% of the trials ( = 62 trials). The first author and the naïve person agreed in 97% of the trials for head turn direction and in 92% of the trials for head position. We used the Kappa test to measure the agreement between the evaluations of the two observers, the naïve person and the first author. A value of 1 indicates perfect agreement and a value of 0 indicates no better than chance agreement (SPSS Statistics 17). The results of the kappa test revealed that reliability was excellent for the head turn direction (kappa = 0.95) and good for the head position (kappa = 0.75).

### Statistical Analysis

Based on all trials, in which subjects showed a response towards the playback stimuli, we calculated an Orientation Index (OI) for each stimulus and subject, according to the formula OI  =  (number right head turns – number left head turns)/(number right head turns + number left head turns). Positive values reflect a right head turn bias – left hemispheric advantage and negative values reflect a left head turn bias – right hemispheric advantage. This index is derived from the Handedness Index [45], which has also been used in studies on auditory laterality (e.g. [26], [27]).

To test for orientation biases in the first session, we used the head turn responses of all subjects (population level) towards each playback category. We tested whether significantly more subjects turned their head to one side than expected by chance, using the Binomial test (e.g. [13], [27]).

To test for consistency of orientation bias we tested also the orientation biases for all sessions together (also on the population level). To do this, we used the *total* number of right and left head turns a subject had made for each playback category. The direction to which the subject showed the majority of head turns for each playback category was then taken as the orientation bias of this subject for this stimulus. We then again tested whether significantly more subjects turned their head to one side than expected by chance, using the Binomial test. In addition we performed a Wilcoxon test to compare the OI between the different playback categories.

To determine effects of the sex-of-receiver we compared the OI of males and females, using a Mann-Whitney-U test for all playback stimuli. To test for an effect of the call type compared the OI's towards tsak calls and trill calls, within each caller category (UO, US and FS; see Figure 1).

To determine effects of familiarity-to-sender, we directly compared the responses of each subject to the call of a familiar sender of the same sex (FS) with the responses to the call of an unfamiliar sender of the same sex (US; Figure 1). To determine effects of sex-of-sender, we directly compared the responses of each subject to the call of an unfamiliar sender of the other sex (UO) with the responses to the call of an unfamiliar sender of the same sex (US).

Using a Mann-Whitney-U test, we tested whether the Orientation Index is affected by the box position. Furthermore, to test for an age effect, we correlated the OI to the age of each subject (Spearman rank correlation). All statistical tests were calculated using SPSS Statistics 17. We found no differences in the OI, between right or left box position (Mann-Whitney-U test: Z≥−1.453, p≥0.146, N_1_≥5, N_2_≥5) for all tested playback stimuli. No significant correlation was found between age and the OI (Spearman test: r_s_≥−0.309, p≥0.245, N≥10) for all tested playback stimuli.

## Results

### Sex-of-Receiver Effect

We found no differences in the OI between males and females, neither in the first session (Mann-Whitney-U test: Z≥−1.291, p≥0.197, N_m_≥5, N_f_≥1 for all tested playback stimuli), nor for the three sessions together (Mann-Whitney-U test: Z≥−1.102, p≥0.270, N_m_≥6, N_f_≥3 for all tested playback stimuli). Since no sex differences were found, we decided to perform all further analyses on the entire subject sample (N = 17), in order to increase the sample size.

### Call Type Effect

No differences were found in the OI towards tsak calls and trill calls, neither in the first session (Wilcoxon test: UO: Z = −1.414, p = 0.157, N = 5; US: Z = −0.577, p = 0.564, N = 4; FS: Z = 0.000, p = 1.000, N = 7), nor for the three sessions together (Wilcoxon test: UO: Z = −0.137, p = 0.891, N = 8; US: Z = −0.740, p = 0.459, N = 11; FS: Z = −0.184, p = 0.854, N = 15). Since no effect of the call type on the orientation biases was found, we decided to analyze the effects of caller characteristics on the orientation biases for trill calls and tsak calls together, in order to increase the sample size.

### Familiarity-to-Sender Effect

**Figure 3:**
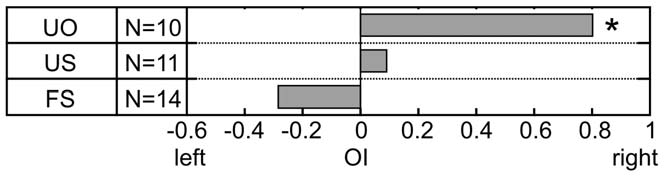
OI index, based on the first session. OI index on the population level, based on the responses towards the three different caller categories, i.e. unfamiliar sender of the other sex (UO) unfamiliar sender of the same sex (US), and familiar sender of the same sex (FS).

In the first session (first playback of every stimulus) no significant orientation bias was found in response to calls of either a familiar, same sex caller (FS; Binomial test: p = 0.424, N = 14) or of an unfamiliar, same sex caller (US; Binomial test: p = 1.000, N = 11; Figure 3 and Table S1). Also, the direction of the OI towards a familiar, same sex caller (FS) did not differ significantly from the direction of the OI towards an unfamiliar, same sex caller (US; Wilcoxon test: Z = −1.127, p = 0.260, N = 12).

Based on the three sessions together, also no significant orientation bias was found in response to calls of either a familiar, same sex caller (FS; Binomial test: p = 0.180, N = 14) or of an unfamiliar, same sex caller (US; Binomial test: p = 0.454, N = 16; Table S2). Also, the direction of the OI towards a familiar, same sex caller (FS) did not differ significantly from the direction of the OI towards an unfamiliar, same sex caller (US; Wilcoxon test: Z = −0.045, p = 0.964, N = 16).

### Sex-of-Sender Effect

In the first session a significant right orientation bias was found in the response to calls of an unfamiliar, other sex caller (UO; Binomial test: p = 0.021, N = 10), but not of an unfamiliar, same sex caller (US; Binomial test: p = 1.000, N = 11; Figure 3 and Table S1). Also, the direction of the OI towards an unfamiliar, other sex caller (UO) showed a (non-significant) tendency to differ from the direction of the OI towards an unfamiliar, same sex caller (US; Wilcoxon test: Z = −1.823, p = 0.068, N = 10).

Based on the three sessions together, we found a significant orientation bias in the response to calls of an unfamiliar, other sex caller (UO; Binomial test: p = 0.021, N = 16), but not of an unfamiliar, same sex caller (US; Binomial test: p = 0.454, N = 16; Table S2). In addition, we found that the OI towards calls of an unfamiliar, other sex caller (UO), differed significantly from the direction of the OI towards calls of an unfamiliar, same sex caller (US; Wilcoxon test: Z = −2.278, p = 0.023, N = 16).

## Discussion

We found that auditory laterality of gray mouse lemurs towards conspecific communication calls was not affected by the sex-of-receiver, call type, or by familiarity-to-sender. However, we did find evidence that auditory laterality in gray mouse lemurs was affected by the sex-of-sender. This effect was also consistent over time, since the results of the first session were confirmed by the results of the three sessions together.

In contrast to the previous study by Scheumann and Zimmermann [27], we found no effect of the sex-of-receiver. In the previous study only male mouse lemurs showed a right orientation bias towards tsak calls and short whistle calls. The difference between these findings can be explained by the fact that in the previous study both males and females were exposed only to female calls. Since in the present study the subjects showed no orientation bias for the same sex calls (US), the results of the previous and present study match. Thus, based on the present findings, we can now conclude that this sex difference was not based on perceptual differences between the sexes, but due to a specific laterality for perceiving the other sex.

In contrast to the previous study [27], we found no effect of the call type in the present study. In the previous study, trill calls elicited no orientation bias in males, whereas tsak calls did. Although we did not discuss the results for males separately in this study, the orientation biases of males towards tsak calls and trill calls did not differ from each other (in both cases a tendency towards a right orientation bias was found). This discrepancy between the previous and present study can also be explained by the use of stimuli from different contexts: whereas the stimuli in the previous study were recorded in the field and in a female sleeping group context, the present stimuli were recorded from the breeding colony in a laboratory setting and in a female-male context. These latter stimuli might therefore be more relevant to our subjects.

No familiarity-to-sender effect was found in the orienting asymmetries of gray mouse lemurs towards conspecific communication sounds. These findings contradict the findings in horses [33], starlings [32], zebra finches [34], and Campbell's monkeys [9]. Conversely, in vervet monkeys also no familiarity-to-sender effect was found on the lateralized processing of conspecific communication sounds [21]. Thus, the existence of a familiarity-to-sender effect on the lateralized processing of conspecific communication sounds in primates remains uncertain and requires further examination. It might be that the human lateralized auditory processing of familiar voices [9], [10] evolved late in primate evolution.

On the other hand, we found an effect of the sex-of-sender on the orientation biases. As far as we know, this is the first study to have focused on such an effect in non-human animals. Because our subjects were tested in the breeding season, our results may be influenced by sexual motivation. Our results might indicate a left hemisphere dominance in sexual behavior in this nocturnal primate, which is in line with the findings of a right eye preference in courtship approach by poeciliid fish [40], but not in line with findings of a left eye preference in courtship approach by black winged silts [39]. Also, our results suggest that a sex-of-sender effect on lateralized auditory processing in humans [38] might have evolved from the lateralized processing of calls of senders of the other sex in early primates. Still the effect of the sex of the sender differs between gray mouse lemurs and humans. Whereas our results on gray mouse lemurs showed a left hemisphere dominance for processing calls of the other sex, humans showed a right hemisphere dominance for processing female voices and a left hemisphere dominance for processing male voices, irrespective of the sex of the listener. Therefore, it seems that the sex-of-sender effect on auditory laterality may have changed during primate evolution. However, more studies on different primate species are necessary to get a clearer picture on the pattern of evolution.

An increasing number of studies have indicated that the mechanisms behind auditory laterality are complex and cannot be explained only by a left hemisphere specialization for conspecific communication, based on temporal cues (e.g. [2], [13], [14]). So far, several studies on humans and non-human animals have shown that conspecific communication is not always processed with a left hemisphere dominance, but can be additionally affected by emotional valence (e.g. [8], [26]) and familiarity to the sender (e.g. [9], [32], [33]). In addition, our present findings suggest that the sex of the sender can also affect auditory laterality, at least in gray mouse lemurs.

All in all, our study confirms the previous findings of auditory laterality in this ancestral primate [27], suggesting that in early primate evolution auditory laterality is present for some conspecific communication calls (as in some non-primate vertebrates; [24], [25]), but not all (in contrast to some other primates; [12], [14]).

Thus, we found evidence for an effect of caller characteristics on the lateralized auditory processing of conspecific communication calls in gray mouse lemurs in the form of a sex-of-sender effect. These findings imply that future research on the evolution of primate auditory laterality should not only explore for effects of conspecifity, communicative significance and emotional valence, but should also take into account acoustically conveyed caller specific information, such as sex.

## Supporting Information

Table S1 Number of animals that turned their head right, left, or not for the different playback categories, in the first session.
(0.03 MB DOC)

Table S2 Number of animals that turned their head more to the right, left, or equally to both sides (ambivalent) for the different playback categories, based on all 3 sessions.
(0.03 MB DOC)

## References

[1] Ecklund-Flores L, Turkewitz G (1996). Asymmetric head turning to speech and nonspeech in human newborns.. Dev Psychobiol.

[2] Belin P, Zilbovicius M, Crozier S, Thivard L, Fontaine A (1998). Laterality of speech and auditory temporal processing.. J Cogn Neurosci.

[3] Bethmann A, Tempelmann C, De Bleser R, Scheich H, Brechmann A (2007). Determining language laterality by fMRI and dichotic listening.. Brain Res.

[4] Zatorre RJ (1988). Pitch perception of complex tones and human temporal-lobe function.. J Acous Soc Am.

[5] Warrier CM, Zatorre RJ (2004). Right temporal cortex is critical for utilization of melodic contextual cues in a pitch constancy task.. Brain.

[6] Pulvermüller F, Kujala T, Shtyrov Y, Simola J, Tiitinen H (2001). Memory traces for words as revealed by the mismatch negativity.. NeuroImage.

[7] Yasin I (2007). Hemispheric differences in processing dichotic meaningful and non-meaningful words.. Neuropsychologia.

[8] Altenmüller E, Schürmann K, Lim VK, Parlitz D (2002). Hits to the left, flops to the right: different emotions during listening to music are reflected in cortical lateralisation patterns.. Neuropsychologia.

[9] Basile M, Lemasson A, Blois-Heulin C (2009). Social and emotional values of sounds influence human (*Homo sapiens*) and non-human primate (*Cercopithecus campbelli*) auditory laterality.. http://dx.doi.org/10.1371/journal.pone.0006295.

[10] Van Lancker D, Kreiman J (1987). Voice discrimination and recognition are separate abilities.. Neuropsychologia.

[11] Zatorre RJ, Belin P, Penhune VB (2002). Structure and function of auditory cortex: music and speech.. Trends Cogn Sci.

[12] Hauser MD, Andersson K (1994). Left hemisphere dominance for processing vocalisations in adult, but not infant, rhesus monkeys: field experiments.. Proc Natl Acad Sci USA.

[13] Hauser MD, Agnetta B, Perez C (1998). Orienting asymmetries in rhesus monkeys: the effect of time-domain changes on acoustic perception.. Anim Behav.

[14] Ghazanfar AA, Smith-Rohrberg D, Hauser MD (2001). The role of temporal cues in rhesus monkey vocal recognition: orienting asymmetries to reversed calls.. Brain Behav Evol.

[15] Poremba A, Malloy M, Saunders RC, Carson RE, Herscovitch P (2004). Species-specific calls evoke asymmetric activity in the monkey's temporal poles.. Nature.

[16] Petersen MR, Beecher MD, Zoloth SR, Moody DB, Stebbins WC (1978). Neural laterality of species specific vocalisations by Japanese Macaques (*Macaca fuscata*).. Science.

[17] Beecher MD, Petersen MR, Zoloth SR, Moody DB, Stebbins WC (1979). Perception of conspecific vocalisations by Japanese macaques.. Brain Behav Evol.

[18] Petersen MR, Zoloth SR, Beecher MD, Green S, Marler PR (1984). Neural laterality of vocalizations by Japanese macaques: communicative significance is more important than acoustic structure.. Behav Neurosci.

[19] Heffner HE, Heffner RS (1986). Effect of unilateral and bilateral auditory cortex lesions on the discrimination of vocalizations by Japanese macaques.. J Neurophysiol.

[20] Böye M, Güntürkün O, Vauclair J (2005). Right ear advantage for conspecific calls in adults and subadults but not infants, California sea lions (*Zalophus californianus*): hemispheric specialization for communication?. Eur J Neurosci.

[21] Gil-da-Costa R, Hauser MD (2006). Vervet monkeys and humans show brain asymmetries for processing conspecific vocalizations, but with opposite patterns of laterality.. Proc R Soc B.

[22] Teufel C, Hammerschmidt K, Fischer J (2007). Lack of orienting asymmetries in Barbary macaques: implications for studies of lateralized auditory processing.. Anim Behav.

[23] Ghazanfar AA, Hauser MD (1999). The neuroethology of primate vocal communication: substrates for the evolution of speech.. Trends Cogn Sci.

[24] Ehret G (1987). Left hemisphere advantage in the mouse brain for recognizing ultrasonic communication calls.. Nature.

[25] Palleroni A, Hauser MD (2003). Experience-dependent plasticity for auditory processing in a raptor.. Science.

[26] Siniscalchi M, Quaranta A, Rogers LJ (2008). Hemispheric Specialization in Dogs for Processing Different Acoustic Stimuli.. http://dx.doi.org/10.1371/journal.pone.0003349.

[27] Scheumann M, Zimmermann E (2008). Sex-specific asymmetries in communication sound perception are not related to hand preference in an early primate.. BMC Biol.

[28] Vallortigara G, Andrew RJ (1991). Laterality of response by chicks to change in a model partner.. Anim Behav.

[29] Deng C, Rogers LJ (2002). Social recognition and approach in the chick: laterality and effect of visual experience.. Anim Behav.

[30] Zucca P, Sovrano VA (2008). Animal laterality and social recognition: Quails use their left visual hemifield when approaching a companion and their right visual hemifield when approaching a stranger.. Cortex.

[31] Peirce JW, Leigh AE, Kendrick KM (2000). Configurational coding, familiarity and the right hemisphere advantage for face recognition in sheep.. Neuropsychologia.

[32] George I, Vernier B, Richard JP, Hausberger M, Cousillas H (2004). Hemispheric specialization in the primary auditory area of awake and anesthetized starlings (*Sturnus vulgaris*).. Behav Neurosci.

[33] Basile M, Boivin S, Boutin A, Blois-Heulin C, Hausberger M (2009). Socially dependent auditory laterality in domestic horses (*Equus caballus*).. Anim Cogn.

[34] Cynx J, Williams H, Nottebohm F (1992). Hemispheric differences in avian song discrimination.. Proc Natl Acad Sci USA.

[35] Petkov CI, Kayser C, Steudel T, Whittingstall K, Augath M (2008). A voice region in the monkey brain.. Nature Neurosci.

[36] Rukstalis M, French JA (2005). Vocal buffering of the stress response: exposure to conspecific vocalizations moderates, urinary cortisol excretion in isolated marmosets.. Horm Behav.

[37] Sproul C, Palleroni A, Hauser MD (2006). Cottontop tamarin, *Saguinus oedipus*, alarm calls contain sufficient information for recognition of individual identity.. Anim Behav.

[38] Landis T, Buttet J, Assal G, Graves R (1982). Dissociation of ear preference in monaural word and voice recognition.. Neuropsychologia.

[39] Ventolini N, Ferrero EA, Sponza S, Chiesa AD, Zucca P (2005). Laterality in the wild: preferential hemifield use during predatory and sexual behaviour in the black-winged stilt.. Anim Behav.

[40] Bisazza A, Facchin L, Pignatti R, Vallortigara G (1997). Laterality of detour behaviour in poeciliid fish: The effect of species, gender and sexual motivation.. Behav Brain Res.

[41] Martin RD, Charles-Dominique P, Martin RD (1972). A preliminary field-study of the lesser mouse lemur (*Microcebus murinus*, Miller 1977).. Behaviour and ecology of nocturnal prosimians.

[42] Zimmermann E, Brudzynski SM (2009). Vocal expression of emotion in a nocturnal prosimian primate.. Handbook of Mammalian Vocalization.

[43] Leliveld LMC, Scheumann M, Zimmermann E (submitted) Acoustic correlates of individuality in the vocal repertoire of a nocturnal primate (*Microcebus murinus*).

[44] Wrogemann D, Radespiel U, Zimmermann E (2001). Comparison of reproductive characteristics and changes in body weight between captive populations of rufous and gray mouse lemurs.. Int J Primatol.

[45] Lonsdorf EV, Hopkins WD (2005). Wild chimpanzees show population-level handedness for tool use.. Proc Natl Acad Sci USA.

