# Supplementary material for: Effects of Caller Characteristics on Auditory Laterality in an Early Primate (Microcebus murinus)
Source: PLoS One. 2010 Feb 3;5(2):e9031. doi: 10.1371/journal.pone.0009031 (PMC2815787; doi:10.1371/journal.pone.0009031)
Supplement: Table S1 — Number of animals that turned their head right, left, or not for the different playback categories, in the first session. (0.03 MB DOC) [file pone.0009031.s001.doc]

Table S1: Number of animals that turned their head right, left, or not for the different playback categories, in the first session.

| Playback stimulus | Right turns | Left turns | No turns | Binomial  (p-value) |
| --- | --- | --- | --- | --- |
| UO | 9 | 1 | 2 | 0.021 |
| US | 6 | 5 | 3 | 1.000 |
| FS | 5 | 9 | 0 | 0.424 |
