# Supplementary material for: Effects of Caller Characteristics on Auditory Laterality in an Early Primate (Microcebus murinus)
Source: PLoS One. 2010 Feb 3;5(2):e9031. doi: 10.1371/journal.pone.0009031 (PMC2815787; doi:10.1371/journal.pone.0009031)
Supplement: Table S2 — Number of animals that turned their head more to the right, left, or equally to both sides (ambivalent) for the different playback categories, based on all 3 sessions. (0.03 MB DOC) [file pone.0009031.s002.doc]

Table S2: Number of animals that turned their head more to the right, left, or equally to both sides (ambivalent) for the different playback categories, based on all 3 sessions.

| Playback stimulus | Right turns | Left turns | Ambivalent | Binomial  (p-value) |
| --- | --- | --- | --- | --- |
| UO | 13 | 3 | 0 | 0.021 |
| US | 6 | 10 | 1 | 0.454 |
| FS | 4 | 10 | 2 | 0.180 |
